# Stakeholder Views on the Potential Benefits and Feasibility of an Equestrian Industry-Specific Health, Safety and Welfare Management System

**DOI:** 10.3390/ani14233450

**Published:** 2024-11-28

**Authors:** Meredith Chapman, Kate Fenner, Matthew J. W. Thomas, Kirrilly Thompson

**Affiliations:** 1 School of Health, Medical and Applied Science, Central Queensland University, Rockhampton, QLD 4701, Australia; 2 School of Agriculture and Food Sustainability, The University of Queensland, Gatton, QLD 4343, Australia; kate.fenner@uq.edu.au; 3 Appleton Institute, Central Queensland University, 44 Greenhill Road, Wayville, SA 5034, Australia; matthew.thomas@cqu.edu.au; 4 Flinders Health and Medical Research Institute, College of Medicine and Public Health, Flinders University, Adelaide, SA 5000, Australia; kirrilly.thompson@flinders.edu.au; 5 School of Medicine and Public Health, University of Newcastle, Callaghan, NSW 2308, Australia

**Keywords:** equestrianism, stakeholders, horses, workplace health and safety, welfare management

## Abstract

With the growth of the equestrian industry, risk exposure and the obligation to maintain the health, safety, and welfare of humans and horses remain front and centre. As there has been no apparent reduction in non-fatal human horse-related injuries, we asked industry stakeholders to discuss their current management and risk mitigation practices and highlight potential barriers to improving these processes. Semi-structured interviews were conducted with 20 stakeholders from Australian equestrian work- (*n* = 9) and non-work- (*n* = 11) related organisations to determine the potential benefits and feasibility of adopting an industry-specific health, safety, and welfare (HSW) management system. The International Organisation for Standardisation (ISO) 45001:2018 Occupational Health and Safety Management framework, often used by high-risk industries, guided deductive data coding. Most participants preferred formal HSW management systems and supported improvements in their current HSW practices. Industry gaps included (1) lack of training, education material, and competency; (2) social licence to operate (SLO) regarding horse welfare; and (3) management of and resistance to change. Industry barriers included (1) lack of good governance and (2) poor safety culture related to tradition and human behaviours. Most stakeholders sought further collaboration, HSW guidance materials, and national industry representation opportunities, with supporting the concept of an adaptable industry-specific HSW management system. Further multi-sector equestrian stakeholder engagement is recommended to determine the feasibility of adopting and modifying (ISO) 45001:2018 as a suitable equestrian industry-specific HSW management system.

## Simple Summary

As the equestrian industry experiences diversification and growth, reviewing and improving health, safety, and welfare practices is crucial. Twenty Australian equestrian industry stakeholders were invited to share their views and identify areas for improvement in a qualitative study. Most participants preferred a formal and adaptable management system, increased industry collaboration, national representation, and general guidance. They reported current organisational gaps and barriers to improvement within their specific industry sectors. The consensus was that the environment in which humans and horses interact requires a comprehensive health, safety, and welfare (HSW) management system approach.

## 1. Introduction

Research suggests that most human horse-related injuries and fatalities can be avoided [1,2,3]. With estimates of rider injury frequency rates based on hours of exposure to be approximately one injury per 1000 h [4], solution-focused equestrian stakeholder engagement and research are necessary to determine best-practice health, safety, and welfare (HSW) risk management interventions for human–horse interactions. To date, most of the approaches to improving safety in equestrianism have primarily focused on personal protective equipment (PPE), such as helmets, or horse behavioural indicators, such as those outlined in Dyson’s Ridden Horse Pain Ethogram [5] and Fenner’s Equine Behavior Assessment and Research Questionnaire (E-BARQ) [6]. Further, the risks associated with human–horse interactions are now being compared to the level of risk in some high-risk sports and workplace industry activities such as mining, construction, aviation, and rail and road transport [1,4,7,8]. However, the equestrian industry has not systematically adopted opportunities to improve HSW for humans and horse welfare or explored the range of workplace health and safety (WHS) strategies available that have been implemented in other contexts or industries [1,8,9].

Risk mitigation and safety management in high-risk industries have typically been achieved by organisations adopting (1) an integrated systems thinking approach, focusing on the way parts of the system interact and how they influence each other as a whole [10,11], and (2) formal WHS management systems [12,13]. The critical principle of WHS systems thinking and management is to mitigate risk [14] and prevent incidents or adverse events by identifying management system failures rather than attributing blame to individuals [15].

All workplaces in Australia have obligations and regulated duties under WHS legislation to ensure the HSW of their workers, including volunteers and relevant others [1,16,17,18]. Workplaces typically meet these duties and obligations through formal WHS system management, and the equestrian industry has been slow to follow this lead [1,19,20]. This is of growing concern for equestrian workplaces such as racing stables, cattle farms, mounted constabularies, and veterinary institutions that need more HSW management systems to meet all of their current workplace obligations. Also, some equestrian workplaces may demonstrate a lack of accepted industry standards, complicating liability in equine-related injuries. Further, stakeholders must understand how HSW best-practice standards and duty of care obligations apply to all types of human–horse interactions, including non-work activities, to dissuade them from presumptions that HSW duties only apply to workplace environments. Thus, one of the goals of this study was to explore whether formal regulation, codes of practice, or other standards were considered appropriate by the various stakeholder groups.

Researchers have begun to explore improved risk mitigation interventions [21,22], risk [9,23] and human risk perception [8], HSW management systems [1,24], evidenced-based safety science [25], training methodologies [26], human–horse behavioural interactions [27], and welfare [6,28] as they relate to work and non-work human–horse interactions. However, little is known about which HSW management systems Australian stakeholders implement, the gaps or barriers encountered during implementation, and the HSW improvements they believe are necessary for the equestrian industry. Further, stakeholders have not previously been asked for their views on the benefits and feasibility of the equestrian industry having access to and use of an industry-specific HSW management system.

The current study consulted with Australian equestrian industry stakeholders to obtain information about what they were currently doing in their respective equestrian industry sectors to manage HSW and the gaps and any barriers they identified during HSW implementation. Further, the study sought insight from stakeholders about (a) opportunities for HSW management improvements and (b) the potential benefits and feasibility of an industry-specific HSW management system similar to those used by some high-risk workplaces, as a standard of excellence.

## 2. Materials and Methods 

This study used a qualitative social science research approach of semi-structured interviews to explore the views of equestrian industry stakeholders. Qualitative methods are often used when little is known about a topic, and further exploration or understanding is required [29].

### 2.1. Recruiting Stakeholder Participants

A purposive sampling strategy was used to recruit stakeholders based on a selection of organisations and businesses known to the first and second authors, as well as internet searches of stakeholder groups, forming an initial representative list of 35 prospective stakeholders. The sample comprised work environments that were classified as stakeholders and their workers who received direct financial benefits, such as an equestrian business or sole trader, and non-work environments, which included sporting associations and not-for-profit organisations. Participation was voluntary, and no reimbursement was offered. This led to the identification of 22 participants from various Australian equestrianism stakeholder groups, such as sporting associations, business owners, and horse healthcare service providers, for example, veterinarians and others working with horses.

The first author contacted each stakeholder via a cold-call telephone introduction to discuss the research study and identify a suitable interview participant from the organisation with HSW operational knowledge and policy decision-making capabilities. The inclusion criteria were: participants over 18 years of age with a minimum of two years of experience in their existing role or similar experience in a previous role in the equestrian sector. Twenty industry stakeholder semi-structured interviews was considered an acceptable sample size for qualitative research practice [30]. Further, this sample size provided adequate information to draw conclusions relevant to the research aims and meet the time constraints of one author conducting the interviews, data collection, and analyses.

Two of the approached stakeholders declined, and the remaining 20 participants who showed interest received an introductory email from the first author, together with a research project information sheet and consent form. Following email replies, each stakeholder was offered a follow-up phone call to answer any additional questions about the research project and the interview process. On receiving each participant’s signed interview consent form, an online Zoom meeting was scheduled for an interview with a duration of up to 60 min. Stakeholders were requested to nominate a representative participant who had current influence over key equestrian HSW policy making or parts thereof and implementation within their stakeholder groups. A total of 20 stakeholder participants agreed to take part in the study with similar representation from work-related (*n* = 9) and non-work-related (*n* = 11) equestrian industry stakeholders. Participant roles varied, with broad stakeholder representation from executive officers, safety managers and officers, volunteers, university staff, emergency response personnel, private business owners, and chairs of not-for-profit organisations.

### 2.2. Data Collection 

Researcher-designed online semi-structured interviews commenced following additional verbal consent from each participant to record the interview via Zoom (V.5.8.6) for transcription purposes. Semi-structured interviews gave the interviewer sufficient flexibility to note unanticipated information from each participant without the need for sub-probe questions [31]. Interviews for the present study were organised around the following three key questions:

1. What does (your equestrian sector) currently do to manage health, safety, and welfare?
2. Describe the gaps and areas for improvement (barriers) that (your equestrian sector) have identified when implementing health, safety, and welfare management?
3. Tell me what the equestrian industry as a whole could do (a) to improve health, safety, and welfare management, and then (b) describe the potential benefits and feasibility of an industry-specific HSW management system.

Participants were encouraged to talk freely, sharing their experience and opinions on each question and their specific HSW operational practices. An introductory probe accompanied each interview question, and sub-probes formed the interview guide (see Supplementary Material). However, most participants responded to each of the three key interview questions without additional prompting. Participant interview times averaged 45 min in duration, with a range of 30–90 min.

### 2.3. Analysis

Audio recordings were transcribed verbatim for each interview and subjected to systematic qualitative data analysis (QDA) with the assistance of NVivo (V.14) software. Green et al.’s four-step process of QDA provided flexibility during the examination and interpretation of the data (2007). This process of data analysis included (1) immersion, (2) coding, (3) categorising, and (4) the generation of themes [32]. The first author read all transcripts thoroughly, then after deidentifying the participant transcripts and sorting the initial data content, all transcripts were re-read, attaching notes to comments as required, consistent with the first step of data immersion.

The following two steps of data analysis co-occurred. Step (2) was coding the data, which included the initial sorting and tagging of transcript words, phrases, or paragraphs. Step (3) facilitated categorising the tagged data into various emerging categories with similar content. All categorised data were initially classified into one of the three interview questions using a deductive coding method.

Instead of using thematic analysis for step (4), the International Organisation for Standardisation (ISO) 45001:2018 Occupational Health and Safety Management framework categories [33] were used to code the participant responses further. This included the findings from question one about current stakeholder HSW strategies and question two about HSW gaps and barriers to implementation, as highlighted in step (3). This framework provides industries with a documented categorical process to identify, control, and manage risks and improve WHS performance. Further, the framework highlights best-practice applications for WHS safety management and risk mitigation by using a systematic approach that was more aligned with the aims of the study and a structured approach to coding interview content. The main categories in ISO 45001:2018 include the introduction, organisational context, leadership and worker participation, planning, support, operations, performance evaluation, and improvements. Some sub-categories, such as resources, competence, awareness, communication, and management of change, were included within the category of support and operations due to the complexity of WHS implementation of processes and procedures. Using this framework, we could also determine frequencies for similar HSW strategies currently implemented by equestrian industry stakeholders.

A slightly different analytic strategy was undertaken for question three due to the diverse range of participant comments and identified categories. Initially, deductive coding was used to determine who was for or against the concept of an industry-specific HSW management system. This was followed by an inductive coding method to identify categories associated with part (a) of question three, identifying stakeholder interests in future improvements for the equestrian industry, and part (b), the potential benefits and feasibility of an industry-specific HSW management system. Further, inductive coding was also used throughout the analysis for comments that may provide value but could not be classified using the deductive coding process.

### 2.4. Ethical Statement

The research was conducted following ethics approval from the Central Queensland University Human Research Ethics Committee, approval number 0000023201.

## 3. Results 

### 3.1. Current Stakeholder HSW Management Strategies 

Participants were asked to share the current HSW strategies implemented in their various organisations. All participants were willing to provide details of their HSW management systems and relevant risk mitigation processes and tools. Protecting equestrianism’s social licence to operate (SLO), as further defined by Douglas et al. [34] as ‘representing an intangible, implicit agreement between the public and industry or group’, was a high priority reported by 75% (*n* = 15) of the participants, expressing an urgency to proactively manage horse health, welfare, ethics, and safeguarding. Over half (60%, *n* = 12) of the participants discussed the importance of integrating *the horse* within the scope of their HSW system. Participants from work and non-work stakeholders were aware of the significance of human–horse interactions, with a particular interest in horse welfare and its association with safety outcomes.

*It sounds to me like a lot of it’s driven by the welfare of the horse and the human. Then that reflects over, across into your safety and your health, so they’re not separated. It’s all one model together. *

*(P12-WK)*

A few participants mentioned the environment forming part of the human-horse-environment trio and its contribution to HSW. However, this was not consistent across the interviews. Over half of the participants reported implementing suitable and consistent horse training methods (*n* = 4), desensitisation practices (*n* = 2), and horse assessments (*n* = 6) in their HSW management strategies to ensure horses were suitable (*n* = 7) and fit-for-purpose for their relevant activities. One participant from a non-work stakeholder suggested that horse and property owners should be licensed to have a horse to improve horse welfare and mitigate the dangers associated with some human–horse interactions. (see Table 1).

#### 3.1.1. ISO 45001:2018 Categories: Organisational Context, Leadership and Worker Participation, and Planning

Most participants, 85% (*n* = 17), reported using formal HSW management systems, with over half (60%, *n* = 12) describing these systems as complex and detailed. Concerning mitigation and system implementation, more than half of the participants discussed the importance of having detailed HSW processes (55%, *n* = 11). Some of these included designated HSW responsibilities, accountabilities, policies, and procedures. Some also stated that they were working toward planned management operations.

Industry leadership, accompanied by transparent communication, was reported as an influential factor for effective HSW management. Identifying key players and ambassadors—and as suggested by one participant, considering the role of Gen Z or Gen Alpha influencers—may also be helpful when taking stakeholders on a continuous improvement HSW journey. One participant discussed the importance of future projection planning, including engagement throughout all organisational levels when sourcing leaders or safety champions. Other comments included:

*There’s more communication now I think, and we’ve got some secretaries and committees that are absolutely leading the way. *

*(P4-NWK)*

*Some of the key influencers in our world on Facebook and Instagram are that young cohort, those 18–24-year-old females, they are kicking huge goals. These young Pony Clubbers are becoming influencers around the world. *

*(P16-NWK)*

Participants raised the topic of the scope of HSW management, including relevant legislation, with mixed views from both work and non-work stakeholders. Some had clear, strong viewpoints, others needed clarification, and some preferred that the equestrian industry did not follow a legislated pathway.

*I don’t think we should be trying to have things legislated because then we take control out of the industry. We need to actually lead and say these are the businesses that we approved, accredited are at a standard that we believe is best practice. I don’t see a lot of downsides to keeping people safe and if it’s not done right as well, we’ve got to find that sweet spot where we don’t overregulate.*

*(P6-WK)*

Planning to meet health and safety legislative and operational requirements for insurance purposes was reported as a necessary consideration for work and non-work activities. The development of proactive hazard identification and assessment processes was described as valuable to reducing reactive and unplanned responses. Participants also raised other topics, such as organisational culture, litigation, advocacy, legal advice, and duty of care (see Table 2).

#### 3.1.2. ISO 45001:2018 Categories: Support and Operations

Participants acknowledged that HSW implementation requires support, adequate resources, and transparent operational processes. Training, competency, and ongoing education and training for humans and horses were prominent discussion points for work and non-work stakeholders. Improved facility management where humans and horses interact led to robust discussions around the specific elements associated with risk control. The most common suggestions were the implementation of regular site or facility inspections, followed by managing the environment, external conditions, and interactive human-horse movements (see Table 3).

#### 3.1.3. ISO 45001:2018 Categories: Performance Evaluation and Improvements

Almost three quarters of all participants mentioned the importance of continuously improving HSW management systems (70%, *n* = 14) by monitoring and reviewing the HSW system and data (60%, *n* = 12). Work stakeholders also reported the value of participating in the accident analysis processes and conducting debriefing meetings for workers and relevant others (see Table 4).

### 3.2. Current Stakeholder HSW Gaps and Barriers to Improvement

Numerous high-risk workplaces use gap analysis to identify areas of HSW system improvement [35,36,37,38,39]. This approach highlights gaps and barriers to continuous system improvement and signifies proactive management compared to reactive and sometimes unplanned responses. Gap analysis does not attribute blame but aims to identify HSW management system failures [40,41,42]. The participants in this study highlighted HSW barriers in the categories associated with organisational context, leadership and worker participation, planning, support, and operations.

#### 3.2.1. ISO 45001:2018 Categories: Organisational Context, Leadership and Worker Participation, and Planning

Governance (60%, *n* = 12) and culture (50%, *n* = 10) were the top two barriers to achieving best-practice HSW, as reported by participants. Governance refers to the systems by which an organisation operates, such as finances, operations, risk management, and oversight of the people in control who are held accountable for their decisions and actions. Some participants reported a few desirable characteristics for people managing governance, such as accountability, transparency, efficiency, inclusivity, responsiveness, consensus-oriented, following rules, and being participatory. A lack of good governance was identified as an area for improvement and a barrier by 67% (*n* = 8) of non-work-related and 33% (*n* = 4) of work-related participants.

*I guess governance of different organisations is an issue for me. It’s the people that end up running these organisations that appear to sometimes get this disproportionate power and disproportionate say in how things happen…I’m not sure how that can be improved. *

*(P1-NWK)*

*Support stems from a grassroots level through to a governance level in terms of these…the sport fundamentally needs to be supported by the people in it and the people in it need to understand that in terms of the sport being sustainable for the future. *

*(P3-NWK)*

Organisational and equestrian safety culture associated with traditional methodologies and the culture of risk-tolerant behaviour were widely identified by participants as barriers to change as they extend beyond this ISO category to implementation and continual improvement. Both work and non-work participants had similar views about managing culture where it led to detrimental effects on organisations and people, particularly impressionable grassroots sporting members. Further, unsafe traditional cultures that influence human risk-tolerant behaviors and unsafe HSW practices across equestrian sectors were mentioned.

*People just take risks all the time because taking risks is a sign of being macho or strong or brave or whatever, but in the horse world, it doesn’t seem to be an understanding that your life and your family and everybody else is at risk by doing something dangerous is a bad thing.*

*(P8-NWK)*

*I think risk tolerance is a really good point and managing that and the culture and making sure that you can still implement what you need to and get the best outcome.*

*(P10-WK)*

*You will never grow a culture without education…a lot of people stick their head in the sand. Don’t want to know about it.*

*(P14-WK)*

Managing the impacts of the SLO and horse welfare was a concern for 50% (*n* = 10) of the participants, who reported no foreseeable active industry government support or regulatory guidance. One participant proposed more industry leadership and best-practice accreditation instead of government regulation.

*There’s a huge liability for this organisation or for the sport, particularly around social license to operate that the sport afforded because there’s no regulation. Even just the introduction of a simple code of practice or a code of conduct would help regulate those sports.*

*(P11-NWK)*

*Overarching framework policies and procedures, yes it would be nice to have the government or an industry body that’s going to just do that, like the regulator to mining or our high-risk industries. SafeWork Australia and SafeWork New South Wales do a little bit, but they can’t be across everything at the end of the day.*

*(P14-WK)*

Equestrianism is a complex and multifaceted industry [28]. There are many different horse-related disciplines, activities, and services, and some participants spoke about the need for a contextualised HSW framework to meet the diversity of stakeholders. Others mentioned how beneficial it would be to have an HSW framework, but questioned how an overarching management system could work for everyone. Some participants acknowledged that a formal HSW structure and overarching governance were necessary. However, for both work and non-work stakeholders, the geographical spread of operations, skills in HSW, and the inconsistency of current equestrianism practices were all reported as collective gaps associated with HSW.

#### 3.2.2. ISO 45001:2018 Categories: Support and Operations 

This category was related to implementing day-to-day HSW management system operations and processes. The most frequently discussed HSW gaps were (1) training, education, and competency (65%, *n* = 13); (2) equestrianism’s SLO associated with managing horse welfare (50%, *n* = 10); and (3) management of and resistance to change (45%, *n* = 9). Participants were also concerned about accessing suitable training information and risk management tools.

*Some more training tools of some sort. Simple things that they can do are occupational health and safety. I stole my risk management design from mining, and then we adjusted it. (For example) a handbook, when someone’s coming into the equine industry, an outline of what you look for and what you do when you’re going to buy a horse.*

*(P5-NWK)*

Problems surrounding the implementation of HSW, such as people and financial resources, adequate communications, and incident reporting gaps, were raised by 40% (*n* = 8) of the participants.

*If we had more resources, we could do things better and faster than it’s taking us to do them now, but we’re on the right track to tick off all the boxes that we need too.*

*(P16-NWK)*

*Probably a gap we see, actually is giving them the skills to communicate and then have the right sort of communication and getting the message across and giving instructions across in knowing that those instructions have been heard and understood.*

*(P10-WK)*

Several concerns were reported about the need for more consistent and ongoing training (40%, *n* = 8) in equestrianism, the variety of training styles and methods (50%, *n* = 10), and the dissemination of specific HSW information and training (40%, *n* = 8). There was a reported lack of knowledge, safety and risk awareness, and experience (50%, *n* = 10) about the fundamentals of safe horsemanship, care, and management in providing horses with optimal welfare. One pertinent example is that anyone can easily purchase a horse without equestrian experience.

*Just the lack of horsemanship knowledge I think is a big issue and people who are not horsey who don’t, it’s a health and safety issue necessarily.*

*(P16-NWK)*

*We still find a huge gap where we have had procedures and protocols in place for 24 months. And people still are not aware of them.*

*(P3-NWK)*

*We’ve got people that are sitting on essentially ticking time bombs, getting around operating far above their capability, because they don’t necessarily have the ability to connect two and two and actually identify the risks.*

*(P11-NWK)*

Varying literacy levels were identified as a training barrier, as were coaching pathways and accreditation, where available. Some participants suggested that additional training in HSW and business management skills (25%, *n* = 5), horse-handling rider assessment, and grading-level skills (30%, *n* = 6) would be beneficial. This was particularly indicated for coaches or trainers who were individual business owners by both work and non-work stakeholders.

*The different levels of education of people are a gap and the accreditation process as a coach. You’ve got people that can do the hands-on paperwork, sign the stuff, get everything done, but actually that experience when you throw them out into the field is not good enough.*

*(P13-WK)*

*There’s a lot of people that are very passionate, a lot of people that have skills in maybe training horses or riding horses, but then actually flowing on to running it in a business sense under the workplace health and safety obligations, there is a big gap there.*

*(P6-WK)*

Concerns regarding equestrianism’s SLO were more commonly highlighted by non-work-related stakeholders (35%, *n* = 7) than work-related stakeholders (15%, *n* = 3). Some participants raised the issue of not having consistent welfare regulations, especially in sporting organisations. Thus, the influence of entrenched outdated traditions and modern advances in social media that enable instant sharing of information and images of poor horse welfare were also highlighted as a concern for the SLO in modern society.

*Today an incident occurs’ in a rural remote town, and it can be on the headline news in a different country. It’s something that wasn’t around like 30 years ago. And we need to ensure that the sport is putting forward a great image and we’re getting participation levels to increase. Otherwise, it won’t sustain and that’s done by managing the safety, health, and welfare aspect of it.*

*(P3-NWK)*

Management of and resistance to change and safety culture appeared to be interconnected when participants talked about gaps or barriers associated with HSW. For example, some equestrian organisations demonstrated minimal evolution and continued to hold onto historical practices. Comments such as, *‘if it isn’t broke, why fix it’* or *‘we’ve done it this way for so long and not got hurt, so why change now?’* were mentioned. For others, trying to keep up to date with current practices and managing changes, legal obligations, social pressures, and economics were considered difficult. Some participants suggested effective change may result from improved communications and viewing negative opinions and organisational gaps as opportunities for proactive improvements.

*There’s a lot of perceived downsides, people are not understanding all of the other benefits. And I think that’s the risk, we’ve got to make sure that we communicate the upsides well, because this is now looking at economic sustainability and career and employment, sustainability and all of these affect risk.*

*(P6-WK)*

One participant discussed the opportunity to close the gap in negative public perceptions around horse welfare, which can impact equestrianism’s SLO. This participant suggested promoting the positive benefits of humans interacting with horses, providing them with adequate horse welfare, and educating the public and equestrianism critics. While the following statement is one participant’s suggestion, and it may not reflect the views of many in the industry, it remains indicative of a traditionally held belief that is unlikely to progress equestrianism’s SLO.

*…if it [the horse] allows itself to be trained, it [the horse] actually gains so much more. It [the horse] gets to travel around, it [the horse] gets cared for, it [the horse] gets vet treatment. It [the horse] now has a purpose to its [the horses] life, not just running around in the paddock or in the bush, living and dying, living breathing, and dying.*

*(P6-WK)*

Another participant discussed the need for consistent safety communications to prevent incidents and accidents, whilst also advocating the benefits of post-incident recovery management for human or horse injuries and fatalities. A lack of post-incident follow-up management was identified explicitly in the non-work environments associated with sporting stakeholder groups.

*Here’s a real area of concern in terms of the information that’s being communicated. Now, how that plays in terms of communication and education is information that our coaches, that our officials are providing to young riders or to inexperienced riders is really inconsistent.*

*(P11-NWK)*

*Once the sirens have been turned off and the patient’s gone to hospital, our focus tends to stop there. We don’t see the emergency as continuing in that recovery phase. So, I think there’s opportunity to improve, particularly when we talk about the welfare of individuals.*

*(P11-NWK)*

### 3.3. Benefits and Feasibility: Equestrian-Specific HSW Management System 

Overall, there was consensus among the stakeholder responses (85%, *n* = 17) on the importance and benefits of equestrianism having access to an industry-specific, adaptable, and integrated HSW management system.

*It’s not just beneficial. It’s, critical for the future of owning and managing horses in Australia.*

*(P1-NWK)*

*That’s probably the big thing we’re saying is we need an integrated industry vertical.*

*(P6-WK)*

*I think any kind of best practice guide in relation to equine industry would be amazing.*

*(P19-WK)*

*It would be great to have an overarching framework. It doesn’t exist yet.*

*(P9-WK)*

However, three (15%) participants commented on difficulties they envisaged within their industry sectors associated with an industry-specific HSW management system and the feasibility of its implementation. These participants were concerned about additional staffing, resources, tradition, bureaucracy, and compliance.

*I guess, pushback that you get, especially dealing with some people in the horse industry that have been in the horse industry for years, they just think it won’t happen.*

*(P19-WK)*

*It’s just another administration and bureaucratic level…I guess my concern is that it may be there just start out as a document that’s recommendations or something and the next thing it becomes rules that it’s just another layer of compliance (P18-NWK). We’ve got to have frameworks that look at suitability, not just, we tell things you must do.*

*(P6-WK)*

Two participants suggested that improvements in HSW management systems would create new opportunities to consolidate existing resources and request further industry funding from sources such as Sport Australia, the Australian Institute of Sport, and the Australian Sports Commission.

*The member groups would all willingly contribute to something like that. We all work in different ways to achieve an outcome…I think everyone would have some input.*

*(P5-NWK)*

*Apparently, (it) has a fantastic board in place that has been incredibly supportive of the future of health, safety, and welfare in the equestrian sector. And that is a bright new life to have, but it needs to continue building on that foundation.*

*(P3-NWK)*

In response to improving HSW for the equestrian industry, 55% (*n* = 11) of the participants, with similar representation from work and non-work stakeholders, suggested that adaptable and best-fit HSW management systems would be valuable to their industry sectors. Participants commented on important features such as (a) information being simple and easily understood, (b) having access to manuals and training tools, (c) the HSW industry being relevant and generic in principle, (d) guidance in practical industry implementation, and (e) accountability factors being included. They emphasised that these would need to be adaptable.

*To be able to have that flexibility within the framework that allows for the risk of the task, you can take into account the experience as well.*

*(P10-WK)*

*I think it’s almost like a step on from that as a bare minimum. Do this, that and the other, and take steps to do this form of guidance, I think would be really beneficial.*

*(P2-WK)*

A consistent approach to HSW management was reported as a principal factor in some work-related (*n* = 5) and non-work-related (*n* = 2) stakeholders. Participants said this would make their job easier in managing HSW matters, especially across various Australian states and territories. Also, industry training and operational practices, with some uniformity regardless of the equestrian activity, would support continuous improvement and reduce attitudes of ‘*she’ll be right’* (P6-WK).

*I don’t disagree that it would bring a level of consistency into the industry, into the mix, across all equestrian disciplines, and it probably would relieve the burden of each association having to put the work in and get their own documentation in place if it was.*

*(P17-NWK)*

*We’ve got in terms of guidelines, safe work guides and codes of practice, and that sort of stuff (information) is a good baseline. And I guess perhaps systems that will help implement that across industries will be helpful.*

*(P7-NWK)*

*Having some sort of best practice that’s brought out that it is, these are the rules, that this is the minimum at least that people need to be abiding by, I think would be amazing. It would make my job a lot easier.*

*(P19-WK)*

Many participants (70%, *n* = 14) in this study were interested in the concept of an industry-specific HSW management system and overarching framework. Over half (55%, *n* = 11) of the participants from work and non-work stakeholders agreed that the concept’s purpose should be for guidance and reference only. Participants also suggested five areas for future consideration to support development and implementation: collaboration, integrated HSW, leadership, improved understanding of legislative or other obligations, and national industry representation.

*Even just a bit of a checklist to go, okay, we’re meeting most of what we see as a best practice guideline, not necessarily regulated, but an overarching framework that you could pull information out and build your own. That would be fine.*

*(P18-NWK)*

*I think that’s the thing is having an accountability model that crosses over all our areas that then we tweak [it] for the specific uses. I think the really big thing is we need to unite ourselves. Not looking at our differences, looking at our synergies.*

*(P6-WK)*

Only one participant was familiar with some industry-specific operational HSW management system processes. Other participants sought further knowledge and understanding of HSW management systems, which were thought to be beneficial rather than a deterrent.

Ninety-five percent (*n* = 19) of the participants, from work and non-work stakeholders, suggested the benefits of collaboration, support, and guidance over legislation and national representation in the future of the industry. Participants were keen to share their knowledge and resources following years of individual system development of documentation and processes, suggesting that this would also assist new industry stakeholders.

*I guess if there’s more collaboration in terms of understanding the operating environments and understanding how to find a way to make it interchangeable, that would be good. I think it’s important to have best access to best practice in collaboration and understanding across the industry together in one room.*

*(P7-NWK)*

Participants recognised other benefits of collaboration, such as sharing funding models, training, and coaching tools, working together on new projects, and benchmarking ideas and standards, given that work and non-work industry environments have similar requirements.

*It’s that opportunity for that cross-industry exchange of ideas.*

*(P10-WK)*

*Everyone is aware that we need to be safe, but we need to continually look at what is working and what’s not. And then try and collaborate again.*

*(P9-WK)*

Finally, participants were interested in exploring national industry representation. However, instead of the term/concept of a ‘regulator’, participants suggested stakeholder representation would be better comprised of a ‘peak body’, ‘coordinator’, ‘governing body’, or similar that provides oversight of the equestrian industry. Overall, participants were optimistic about the potential industry benefits of having access to auditing, licensing, national standards, traceability for horses, and opportunities for shared services.

*I think for an organisation like ours, it honestly wouldn’t worry me to have someone come in and check and make sure that we’re doing things right. Because I know that we are, and we don’t have a problem with being compliant because our staff safety is the most important thing. I think a regulator would actually be a good thing.*

*(P19-WK)*

*It would be nice to have this governing body that could be enforcing it, because I think it will need to be enforced [to be] safer all around, but you would hope to start with it not enforced; it’s something that people want to come along to. I think it’s going to have to be government body driven. It’s all about better unity, [it’s a] huge benefit to moving forward to keep everyone safe.*

*(P14-WK)*

## 4. Discussion 

The findings of this study highlight that many equestrian industry stakeholders have reasonable knowledge about HSW, are interested in opportunities for improvement, and are concerned about the sustainability of equestrianism. Stakeholder representatives were also aware of their multi-sectorial differences in work and non-work organisations and shared operational gaps and barriers during equestrian-related activities. Stakeholders were confident about what they believed would enable best-practice HSW management, including how to overcome perceived barriers. These findings support the feasibility of an equestrian industry-specific HSW management system and framework, with some reported barriers being potential enablers, such as governance, leadership, and culture.

### 4.1. Unpacking the Gaps, Barriers, and Enablers 

Limited industry governance, leadership, and poor safety culture were reported as widespread concerns for work and non-work stakeholders. However, a lack of internal organisational governance was more apparent for non-work equestrian stakeholders, i.e., during sports and leisure activities. This may be due to unpaid and time-poor volunteer workers, multiple committees, and challenges in securing consistent sports management leadership due to competing higher-paid job market opportunities. Further, the Australian equestrian industry does not currently have overarching governance or national industry representation [1,28], whereas other high-risk workplaces do have such facilities. For example, aviation has the Civil Aviation Safety Authority (CASA) [43] as a government regulator, and railways have partnered with the Rail Industry Safety and Standards Board (RISSB), a non-government member-funded organisation [44].

In this study, equestrian industry participants shared views of limited governance coupled with the need for structured and effective equestrian industry leadership and improved culture. These participant views were reported despite some existing equestrian-related organisations, such as the Federation Equestre Internationale (FEI), Equestrian Australia (EA), the Australian Veterinarian Association (AVA), and the Australian Horse Industry Council (AHIC), which provide some guidance and governance for their respective sectors through a management team and board of directors. However, organisational oversight is only available to a few equestrian industry sectors and is not consistent across all work and non-work stakeholder operations.

The equestrian stakeholder industry concerns reported in this study support the concerns of some researchers who have also highlighted some negative impacts of poor governance, leadership, and culture on HSW management practices and their influence on the sustainability of the equestrian industry [19,45,46,47,48]. The literature suggests that the equestrian industry has limited knowledge in understanding the intrinsic links between governance, leadership, and culture, unlike the aforementioned high-risk industries that have successfully applied governance and monitoring of organisational leadership and safety culture [49,50]. Thus, effective leadership is necessary to ensure good governance over HSW management systems, resulting in an enriched and supportive safety culture that drives continuous HSW industry improvements.

Some work stakeholders were more familiar with business-related WHS legislative requirements, similar to other high-risk industries, including opportunities for regular work-sector consultation and collaboration [51]. For example, agricultural industry stakeholders reported having access to various national groups, such as the Meat and Livestock Association (MLA) and Farmsafe Australia, where HSW operational resources and education tools are readily available. In comparison, some non-work stakeholders hesitated to consult and seek support from similar industry sectors. An explanation for this reluctance may be due to the competitive nature of equestrian sport, difficulties in sourcing volunteers, reliance upon membership-based participant funding, and a general lack of understanding of not-for-profit HSW accountabilities and responsibilities. However, despite some industry sector differences, this study highlighted substantial stakeholder interest in achieving consistent national industry collaboration. Multi-sector equestrian stakeholders, such as not-for-profit associations, clubs, sole traders (e.g., coaches), and business owners, face similar risks and challenges. Further, human–horse interaction risks are well known to the equestrian industry, with the environmental risk factors being similar regardless of the equestrian activity [52].

Participants believed more time is necessary to plan and work on HSW matters and there needs to be more information sharing, given that many stakeholders operate with volunteers and share similar operational and funding issues. Stakeholders already have access to face-to-face or online national and international seminars and conferences and can become involved in industry project groups to discuss shared issues and topics of interest. For example, some equestrian-specific industry opportunities regarding horse welfare include the British Animal Rescue and Trauma Care Association (BARTA) [53] (with a focus on large animal rescues), biosecurity matters from Animal Health Australia (AHA) [54], the FEI Ethics and Wellbeing Commission [55], and the International Society for Equitation Science (ISES) [56]. However, the problem remains that information about collaborative opportunities is not widely disseminated across all industry sectors. The equestrian industry has no national representation or central information hub to endorse and circulate knowledge, relying instead upon each sector to independently source or share information with other stakeholders. The benefits of nation-wide equestrian industry collaboration and engagement far outweigh the time and costs of industry stakeholders reproducing individual HSW management systems. Thus, with national representation, a central repository or information hub is likely to be advantageous for all sectors of the equestrian industry [53,54,55,56].

Maintaining the equestrian industry’s SLO regarding how human–horse interactions are undertaken and how they are perceived (by outsiders and from within) were concerns for both work and non-work stakeholders. The question of the SLO often arises due to the fact that animals are being involved in sporting interactions for human gain. Non-work stakeholders reported the SLO as a problem, particularly in sport horse activities. This may be because sport horses often attract extensive media exposure from public viewing, from local weekend sports events to the globally televised Olympic Games. Thus, other equestrian sectors, animal welfare organisations, the general public, and activists have frequent opportunities to scrutinise and publicly comment on equestrian activities. By contrast, work-related activities are less likely to be promoted in social and media exposure without prior approval from management.

Training and competency were considered ongoing problems for stakeholders in this study, particularly when accessing relevant and consistent equestrian industry training methods. The equestrian industry is multi-sectoral in work and non-work equestrian activities and multifaceted, having variable requirements to operate safely within each area. In addition, numerous external factors, including legal obligations, WHS, corporate governance, animal health, insurance, finance, workers’ compensation, and the SLO, constantly impact daily operational activities. Therefore, providing industry-wide training and competency programs is impractical and unlikely to occur as there is no current industry governance and national representation. However, the equestrian industry can foster more stakeholder collaboration and share relevant training programs and materials through member-based entities such as AHA, ISES, AHIC, Horse Safety Australia, and Safework Australia. Further, industry collaboration creates opportunities to develop consistent industry-specific HSW management systems and minimal training competencies standards for each industry sector. These risk mitigation and management practices are likely to support a reduction in human horse-related injuries and fatalities.

Some participants in this study expressed concerns that horses continue to be viewed as dangerous and unpredictable, which can explain why some humans have a general acceptance of risk during equestrian-related activities [9]. This is despite accident analysis investigations identifying that this is not always the case, given that humans are the decision makers who manage safer human–horse interactions and guardians of the horses [8,9,57]. An explanation for some of these assumptions that horses are *just dangerous* and the risks are unmanageable may be due to inadequate stakeholder and individual understanding of how to use accident analysis to determine contributing factors and an influential source of documentation that appears on many insurance liability release forms stating that ‘horses are dangerous animals’. Further, a limited understanding of horse ethology, training, and welfare likely supports an acceptance of horse unpredictability and inherent dangerousness [58,59] when humans can improve their ability to know and predict horse behaviour [9]. Beyond these assumptions, research highlights the environment where human–horse interactions occur as a significant causal factor for many accidents [16,60]. Additionally, post-incident investigations [1] and accident analysis models for equestrianism are only just emerging, such as Human Factors and Classification System-Equestrianism (HFACS-Eq) [60] and the Triple-E Model that provides benefits for thoroughbred racing [16]. These tools enable proactive change and identify other manageable HSW opportunities to mitigate risk during human–horse interactions.

#### Including Horses in HSW Management Systems as ‘Working Equids’

Humans use horses for work globally, with Carter [61] estimating that there are over 100 million working equids. Examples include horses used for work-related activities such as mustering livestock, mounted constabulary, equine tourism, and animal-assisted human therapy. Researchers have also investigated horses in the context of working equids, such as horses, donkeys, and mules being used for transportation and farming, particularly to assess their welfare needs [62,63,64,65]. For many years, workplaces such as the agricultural sector and the mounted constabulary referred to horses as ‘living machines’ or ‘equipment’ [66,67]. More recently, a few equestrian stakeholders referred to horses as ‘working plant’ and ‘working animals’, also recognising the importance of animal sentience [68], which supports horses as ‘working equids’ [69] not as objects [70]. Acknowledging animal sentience is the key to ensuring stakeholders understand the value of good horse welfare practices. Thus, stakeholders who recognise the significance of reporting, discuss the importance of, and maintain documented records of maintenance and welfare for each working horse demonstrate practical and diligent horse welfare practices [71]. These systematic HSW practices would go a long way toward protecting the welfare of these working animals. However, they are not currently an industry requirement. Further, some benefits of work stakeholders adopting a more consistent approach to classifying and acknowledging horses as working equids far surpass any perceived disadvantages. Some benefits may include (1) assurance of adequate horse welfare with the value of horses being recognised, communicated, and managed as working and sentient counterparts; (2) enhanced legal and governance protection, such as workload, welfare, and humane treatment standards; (3) improved funding, resources, and educational opportunities to support proactive welfare and improved working conditions; (4) enhanced cultural and economic value whereby traditional, lifestyle, and tourism practices are preserved; and (5) promoting sustainable work practices and economic stability in societies where modern machinery is less accessible.

Horse welfare and ensuring horses have ‘a good life’ is a burning issue for equestrianism globally, with the media now focusing on horse welfare issues more than ever before [72]. With work-related equestrian stakeholders and researchers suggesting that horses should be acknowledged as working equids, they should be included in day-to-day HSW work operations and risk management practices [73,74]. With collaborative equestrian industry acknowledgment and understanding [75,76] and work-related policymakers and regulatory authorities such as Safework Australia endorsing horses as working equids [77,78,79], the equestrian industry is more likely to improve future human–working equid relations. Thus, it will reduce the incidence and severity of human injuries and fatalities while simultaneously improving horse welfare and the SLO of work and non-work stakeholders as a whole.

### 4.2. Supporting Systems Thinking and HSW Management for Equestrianism 

This study found that 85% (*n* = 17) of the participants were currently using various formal HSW management systems, while others were still working toward achieving formal HSW processes and protocols. Since the 1970s, various applications of HSW management, commonly referred to in the literature as occupational health and safety management systems (OHSMS), have been developed and used in workplaces and private and not-for-profit organisations [80]. Management systems vary depending on the operational needs of work or non-work environments. However, they usually include best-practice standards and procedures, guidelines, and audit processes to manage and monitor HSW [81]. Earlier research by Redinger and Levine [82] suggested that 16 primary elements were necessary in any OHSMS model. They recommended that OHSMS should include (1) management commitment and resources, (2) employee participation, (3) occupational health and safety policy, (4) goals and objectives, (5) performance measures, (6) planning and development, (7) OHSMS manual and procedures, (8) training, (9) hazard control, (10) preventative and corrective actions, (11) procurement and contracting, (12) communication, (13) evaluation, (14) continual improvement, (15) integration, and (16) management review. This demonstrates that best-practice OHSMS frameworks are well established. The International Organisation for Standardisation (ISO) 45001:2018 Occupational Health and Safety Management framework is an example of a documented categorical model to identify, control, and manage risks [33], which could be adapted for HSW management in the equestrian industry.

Over half of the stakeholders reported having detailed management systems for training and competency assessments, reporting, record keeping, communication processes, and risk management. As highlighted previously, these are only some fundamental components of a health and safety management system, with a few stakeholders advising that they accessed templates from other non-equestrian workplaces or downloaded them from the Internet. Generally, off-the-shelf HSW management system policies, procedures, risk management protocols, training programs, communication pathways, and monitoring protocols are helpful; however, they require further modification to meet the requirements of equestrian industry-specific activities. For some stakeholders, simply contemplating these HSW modifications and further developing these management systems will likely be difficult without HSW and equestrian expertise. This may explain why there were deficits in some stakeholder HSW management systems, given that the equestrian industry, unlike other work or sport-related organisations, include the horse as an integral component.

Generally, participants were positive during the interview discussions about opportunities for improved planning and management of HSW matters, with both sectors cognisant of the importance of management responsibility and accountability. However, non-work stakeholders sought more clarity and understanding of achieving HSW improvements. This may indicate that some stakeholders have limited access to business modelling and limited finances to implement these fundamental management processes, often relying upon a volunteer workforce. Thus, it was unsurprising that 60% (*n* = 14) of the participants favoured improving their existing HSW management, and 70% (*n* = 14) were interested in the future development of an equestrian industry-specific HSW management system.

All participants in this study acknowledged horses as integral to their industry, with 75% (*n* = 15) expressing their concerns for horse welfare management, ethical human-horse practices, the SLO, and safeguarding horses within the equestrian industry. This included managing horse health, safety, and welfare during daily operations and demonstrating best-practice management for the overall sustainability of the equestrian industry. However, over half of the participants reported difficulties formally reporting, documenting, and implementing some of these processes. This substantiates prior participant comments about standard HSW management systems requiring further adaptation to be relevant to equestrian industry-specific operations. Some examples include developing or resourcing protocols to assess and grade rider competency levels and identifying supervision requirements, equestrian facility inductions, safe operating procedures when handling and riding horses during various activities, and risk awareness training around horses. Whereas most participants were confident in horse ethology, training, and welfare within their respective industry activities, formalising some of these daily practices was reported as challenging. Therefore, it is recommended that Raw et al.’s [69] ‘working equid’ definition as “any equid engaged in physical labour that provides a significant or direct contribution to the economic livelihood, sustenance or support of the owner/user’s family, typically within a low resource setting” be adopted when developing and implementing HSW management systems in equestrian workplaces. Some HSW management practices specific to horses may include monitoring and maintaining records of the horse’s veterinary, farrier, and dental records, procurement processes for purchasing or leasing horses, and breeding and training records. Also, documenting assessments during the various stages of the horse’s training regime and behavioural responses, as can be achieved using the Equine Behaviour Assessment and Research Questionnaire EBARQ [6], along with horse and rider assessment allocation, would demonstrate best-practice HSW management. By comparison, for sport and leisure horses (non-work), HSW management is usually the responsibility of horse owners or their designated guardians. However, implementing similar work-related horse welfare monitoring and management practices would support the equestrian industry’s SLO. Further, sports and leisure riding facilities that organise events, training days, and competitions are also responsible for ensuring their participants know the risks associated with equestrian sport and riding. For example, managing events and volunteers, checking that the riding or activity environment is safe, maintaining facilities, and providing adequate medical support.

This study demonstrates that equestrian industry stakeholders still have questions about developing HSW management systems to meet their relative HSW obligations for humans, provide best-practice horse welfare, and ensure the equestrian industry’s SLO is not threatened. Given the unique risks inherent in and associated with human horse-related activities in work and non-work environments, this may explain some of the industry’s current HSW management systems gaps and support stakeholder requests for further guidance and support. It is worth noting that some equestrian-specific HSW management tools and information are already available. For example, SafeWork NSW Australia developed an industry-specific Code of Practice, the ‘Guide to managing risks when new and inexperienced persons interact with horses’ [83], accompanied by an information sheet about ‘Working safely with horses’ [84] and video ‘Working with horses safety alert’ [85]. Worksafe Victoria also produced the ‘Guidebook: Horse Stables and Track Riding Safety’ [86]. Other associations, such as the Australian Horse Industry Council have a ‘HorseSafe Code of Practice’ and resources (AHIC, 2009), and Horse Safety Australia offers resources, training programs and facility accreditations [87]. However, this information was not known by all the participants in this study, which signifies a lack of industry awareness of resources and highlights the importance of having centralised, nationally endorsed, accessible and cost-effective education and training for equestrian industry stakeholders.

Less than half of the participants interviewed expressed mixed views about legislative health and safety regulations and how these fit into the equestrian industry. Some work-related stakeholders did not favour legislation and overregulation. A few participants reported they preferred to have more control over the equestrian industry and how it functions, and others advised they were not fully aware of their obligations due to limited guidance. However, legislative HSW obligations are mandatory for all workplaces, being the responsibility of persons with control over daily operations and are held accountable for mitigating and managing risk [88,89]. These responses may indicate that some of the stakeholders are not fully aware of their legal WHS obligations per the Australian government regulations for Persons Conducting a Business or Undertakings (PCBU) to keep people safe at work, including volunteers [90]. Thus, one of the benefits of more formal regulation or standards relating to the equestrian industry would be a new imperative to change HSW practices due to the increased liability of business owners in the event of an injury or accident. Further, other stakeholders (not-for-profit organisations) also have similar HSW obligations as any other employer to their workers, with company directors and committee members of incorporated and unincorporated associations being responsible for managing HSW [91]. Workplace Health and Safety legislation does not apply to not-for-profit organisations deemed entirely volunteer associations [92]. That being said, several successful equestrian industry prosecutions in Australia have demonstrated breaches of HSW systems management responsibilities and stakeholders’ accountabilities. For example, the sport-related case-law findings [93] and the workplace prosecution [94] highlighted multiple HSW management systems failures, including unstable changes to ground surfaces, limited supervision, and failures in horse rider-skill match. Therefore, it is likely that some of the stakeholders represented in this study were not fully aware of their legal HSW obligations, duty of care responsibilities and accountabilities within their various equestrian industry sectors. More education on these obligations and industry engagement will likely improve stakeholder knowledge and motivate the adoption of a formal industry-specific HSW management system. Nevertheless, a lack of knowledge does not dismiss the duty of care and responsibility for work and non-work equestrian stakeholders to source this information and ensure responsible human-horse activities occur.

**Figure 1:**
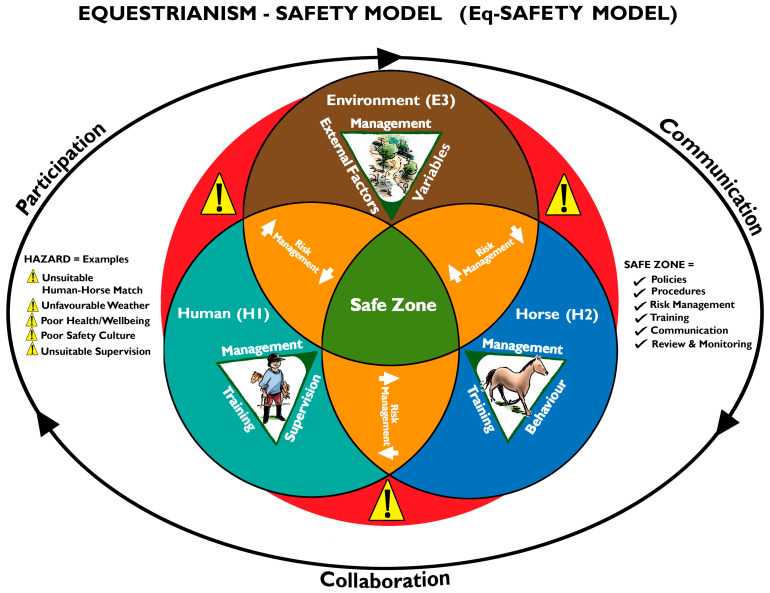
Eq-Safety Model: Design of systems thinking HSW management and risk mitigation process for equestrianism, including continuous stakeholder communication, collaboration and participation.

Participants expressed the need for an integrated approach to managing human-horse-environment risk. However, some participants found it difficult to describe what this concept would look like and how the three HSW elements would interrelate. High-risk workplaces, such as the oil and gas, mining, construction, and chemical industries, are also inherently hazardous and typically use integrated health, safety, and environment (HSE) management systems, accident analysis tools, and safety audits to manage and mitigate work risks. The recently developed and validated accident analysis tool Human Factors and Classification System-Equestrianism (HFACS-Eq) [60] allows the equestrian industry to analyse systems and human failures following human-horse incidents and accidents. Further, HFACS-Eq could also be used by all stakeholders as a proactive risk mitigation audit tool to identify potential human, horse, environmental, and organisational system failures, implementing preventive HSW system risk controls to prevent human horse-related incidents and fatalities. As suggested by participants within this study and following analysis of equestrian industry gaps, multi-sectorial industry guidance, further education, and consideration for national equestrian industry representation should be an ongoing industry priority. To assist equestrian stakeholders and researchers [1,9,16,21,24,95], the Equestrianism Safety Model (Eq-Safety Model) has been developed to help build stakeholder capacity in thinking about, understanding, and operationalising a systems approach to HSW risk mitigation practice and management when applied to their industry, similar to high-risk industries [96,97]. Despite this study’s limitations and small sample size, the Eq-Safety Model should be viewed as a larger generalisation and application of the study findings to further assist stakeholders in planning safer integrated work and non-work equestrian activities. As an educational tool, the model demonstrates safety and risk mitigation systems thinking about the human–horse–environment relationship, each having equal importance when identifying, assessing, and managing risk. That is, it illustrates what an integrated approach to managing human-horse-environment risk looks like and how the three HSW elements interrelate. A brief description of the Eq-Safety model is outlined below (see Figure 1).

#### Equestrianism Safety Model (Eq-Safety Model)

- The outer circle containing the triad represents the significance of stakeholder leadership, guidance, and management, facilitated through effective collaboration, communication, and participation with workers, volunteers, participants, and others.
- Elements within the model, (H1) human, (H2) horse, and (E3) environment have independent operational requirements to achieve optimal status quo. For example, (H1) human requires management processes, training, supervision, and so on.
- The outer red zones, with yellow hazard signs between each element, demonstrate the ever-changing position and vulnerability of triad (H1–H2–E3) interactions. For example, a hazard warning arises for an unsuitable human-horse match (H1-H2) or a hazard warning for a pending change in weather conditions (E3-H2). All require proactive risk management responses.
- The orange zones between each element represent the need to identify ongoing proactive and practical risk management practices relative to all element combinations. For example, pre-event planning and site/facility inspections (H1-E3-H2), supervisory corrections (H1-H2), horse welfare needs (H2-E3), emergency access and egress (H1-E3-H2). The green central safety zone is the state of equilibrium representing safer human–horse interactions. A continual review of systems thinking and safety modelling will reduce human horse-related injuries and fatalities. However, risk management (orange zone) remains ongoing.

## 5. Limitations

This study was limited to 20 participants, with a diverse selection of stakeholder representatives. However, stakeholders from specific industries, such as equine-assisted therapies, thoroughbred racing, and farrier associations, did not participate in this study. Only Australian stakeholder participants were recruited, and further research may benefit the industry in sharing a generic HSW management framework, as suggested in this study, which could be tested globally and include more industry stakeholders. The Eq-Safety Model could provide a means through which to engage more diverse participants in discussions about developing and formalising HSW management systems for equestrian stakeholders and sectors. Involving participants from other countries may also provide insight into how other countries implement and manage HSW, supporting widespread stakeholder collaboration. Participant comments were primarily based on their current HSW management practices. Nevertheless, semi-structured interviews with this sample size provided a depth of understanding about current practices and the feasibility of a HSW management system and framework tailored for equestrianism.

## 6. Conclusions

Most participants in this study were receptive of the future concept of an equestrian industry-specific HSW management system and framework, suggesting that it is feasible. In particular, this study demonstrated stakeholder interest in pursuing opportunities for more collaboration to explore the benefits of an equestrian industry-specific HSW management system framework. Collaboration would also identify education and training tools to support integrative management of human-horse-environment risk to help organisations meet their relative HSW obligations for human safety and horse welfare, thereby protecting the industry’s SLO. As a first step, equestrian multi-sector stakeholder engagement and further collaboration are recommended to formalise a national central educational hub to share equestrian industry-specific HSW management tools, educational programs, research, and relevant government or industry updates. Next, it is imperative to establish or agree upon existing national industry, not-for-profit organisations, or agencies that could develop best-practice industry-specific HSW management and risk-mitigation guidelines for work and non-work stakeholders. For example, Farmsafe Australia or the Australian Horse Industry Council. Therefore, following the establishment of national industry representation, the feasibility of adapting the International Organisation for Standardisation (ISO) 45001:2018 Occupational Health and Safety Management framework [33] as a suitable HSW management system framework with adaptation to include the horse could be investigated. In addition, further national industry engagement with insurance agencies in developing risk mitigation tools and HSW management systems will demonstrate proactive industry commitment and support all stakeholders in obtaining and maintaining business or recreational liability insurance. Despite reported HSW gaps/barriers and growing concerns about the SLO, horse welfare, and poor culture, stakeholders supported guidance and leadership rather than enforcement. This study highlighted current equestrian groups and organisations that provide HSW industry-specific education and resources, as well as the potential benefits of recognising horses as ‘working equids’ for work-related activities. Further, to encourage a systems-thinking approach for stakeholder HSW management, the Eq-Safety Model provides a simple, visual educational tool to contextualise the interrelated elements of humans, horses, and the environment, demonstrating a whole HSW system management approach. Ongoing stakeholder collaboration to further develop this model as a broader industry application would be beneficial. Further, the validated accident analysis tool Human Factors and Classification System-Equestrianism (HFACS-Eq) [60] provides the industry with a tool to analyse pre-event or post-incident or accident HSW systems and human failures, whereby data analysis findings can provide future guidance for industry improvements. Equestrianism in the context of humans interacting with horses in work and non-work-related activities is here to stay. To preserve the memorable history of humans and horses in society and protect the future sustainability of the equestrian industry, we must work together to implement safer systems for humans and improve horse welfare, both of which are essential to reducing future human horse-related injuries and fatalities.

## Figures and Tables

**Table 1:** Frequently identified current stakeholder HSW management strategies and horse-related factors coded from participants’ responses.

Horse Factors			
Current Stakeholder HSW Management Strategies	Work	Non-Work	Total %
Protecting Equestrianism’s SLO: Horse Health, Welfare, Ethics and Safeguarding	7	8	75%
Horses Embedded within the HSW Management System	6	6	60%
Suitable Training, Assessing, Desensitising Horses	6	3	45%
Horse Suitability (fit-for-purpose) for Work and Non-Work Activities	5	2	35%
Dangerous and Risky	2	3	25%

**Table 2:** Frequently identified current stakeholder HSW management strategies using ISO 45001:2018 categories organisational context, leadership and worker participation, and planning to code participants’ responses.

ISO 45001:2018 Categories: Organisational Context, Leadership and Worker Participation, Planning			
Current Stakeholder HSW Management Strategies	Work	Non-Work	Total %
Using Formal Systems	8	9	85%
Designated Responsibilities and Accountabilities	4	8	60%
Hazard Identification and Risk Assessment	6	6	60%
Risk Mitigation and Management Practices	6	5	55%
Policies, Procedures, Rules, and Safety Number 1	4	6	50%
Enforcement and Legislation	4	5	45%
Insurance	2	6	40%
Proactive vs. Reactive HSW Management	3	3	30%
Code of Practice	2	3	25%
Using Simple Systems	3	2	25%

**Table 3:** Frequently identified current stakeholder HSW management strategies using ISO 45001:2018 categories of support and operations and sub-categories (a) resources, competence, and awareness, (b) communication, (c) documented information, and (d) operational planning and control to code participants’ responses.

ISO 45001:2018 Categories: Support and Operations			
Current Stakeholder HSW Management Strategies	Work	Non-Work	Total %
*(a) ISO Sub-category: Resources, Competence, and Awareness*			
Training Processes and Procedures	7	5	60%
Competence	5	5	50%
Lack of Safety/Risk Training, Advice, and Reinforcement	4	6	50%
Ongoing Education	4	4	40%
Horse Suitability and Human Matching	5	3	40%
Specific HSW Training	4	4	40%
Assessing, Grading, and Levels of Riders	2	4	30%
Supervision and Coach Ratios	3	2	25%
Basic Business and Adaptable Safety Education	2	3	25%
*(b) ISO Sub-category: Communication*			
Incident Management, Reporting Including Near Miss, and Data Analysis	6	5	55%
Importance of Communications	3	3	30%
Site or Facility Inductions	4	2	30%
Promoting Discussions	4	2	30%
Regular Updates and Comms	1	4	25%
*(c) ISO Sub-category: Documented Information*			
Detailed System Management	7	5	60%
Documentation and Record Keeping	5	4	45%
Standard or Safe Operating Procedures	5	3	40%
*(d) ISO Sub-category: Operational Planning and Control*			
Safety Equipment and Fit-For-Purpose	6	6	60%
General Facilities Management	3	7	50%
Risk Controls, Including Rules	4	6	50%
Types of Injuries During Human–Horse Interactions, Imminent Danger	4	4	40%
Staff Welfare, Wellbeing, Mental Health, and Code of Conduct	4	4	40%
Personal Protective Equipment (PPE)	2	3	25%
Including Volunteers and Spectators		5	25%
Incident Response, Minimal Medical Support, and Standards	1	4	25%
Environment and Surroundings	2	3	25%

**Table 4:** Frequently identified current stakeholder HSW management strategies using ISO 45001:2018 categories performance evaluation and improvements to code participants’ responses.

ISO 45001:2018 Categories: Performance Evaluation and Improvements			
Current Stakeholder HSW Management Strategies	Work	Non-Work	Total %
Continuous Improvement	7	7	70%
Monitoring and Reviewing HSW Systems and Data	5	7	60%
Incident Causation Analysis and Post-Incident Debriefing	5	0	25%

## Data Availability

The data supporting this study’s findings are not publicly available due to the privacy of stakeholders or ethical restrictions.

## Supplementary Materials

The following supporting information can be downloaded at: https://www.mdpi.com/article/10.3390/ani14233450/s1, Document S1: Participant Interview Guide.
