# Supplementary material for: Stakeholder Views on the Potential Benefits and Feasibility of an Equestrian Industry-Specific Health, Safety and Welfare Management System
Source: Animals (Basel). 2024 Nov 28;14(23):3450. doi: 10.3390/ani14233450 (PMC11640081; doi:10.3390/ani14233450)
Supplement: Supplementary file 1 [file animals-14-03450-s001.zip › animals-3245487-supplementary.pdf]

**Stakeholder views on the potential benefits and feasibility of an equestrian industry-specific Health, Safety and Welfare Management System. Participant Interview Guide - Supplementary Material**

Questions, Main Probe (Introduction) and Sub-probes

**Q1. What does (<equestrian sector>) currently do to manage Health, Safety and Welfare?**

I am interested in understanding what < equestrian sector > currently does to manage and meet its Health, Safety and Welfare obligations for participants and their horses?

- Have you developed your own systems?
- How do you think these practices have evolved?
- Tell me what guidance has <specific equestrian sector> sourced from industry to assist with Health, Safety and Welfare management?
- Do you separate Health Safety and Welfare, or do they overlap? Do you perhaps, take Health, Safety together and address Welfare differently (If at all)?
- Are these practices more informal or formal?
- Describe what factors influence or contribute to your current practices? (e.g., legislation, industry performance, accreditation, funding)
- How do you currently manage implementation?
- How do/Do you monitor adherence to your guidelines?
- How do you monitor the effectiveness of your current practices for risk mitigation?

**Q2. Describe the gaps and areas for improvement (barriers) that (<equestrian sector>) have identified when implementing Health, Safety and Welfare management?**

We know equestrianism is *high-risk,* and risk management is a critical component for <specific equestrian sector> to promote and maintain healthy and safe human-horse interactions for the benefit of both human and horse. Can you identify any gaps or areas for improvement (barriers) within <specific equestrian sector>?

- Can you describe the various levels of *risk to human participants* within <specific equestrian sector>?
- Can you describe the various levels of *risk to horse welfare* within <specific equestrian sector>?
- Do you think more needs to be done in this area?
- What do you think we can learn from other *high-risk* industries? (for example, training opportunities, compliance or conformance strategy, processes for monitoring risk)
- What difficulties has <specific equestrian sector> identified in risk management?
- What seems to be working well?
- Explain why these items are working well?
- Tell me what support does <specific equestrian sector> need to maintain best practice standards?

- What is stopping <specific equestrian sector> from achieving best practice Health, Safety and Welfare management?

**Q3. Tell me what the equestrian industry as a whole could do to a) improve Health, Safety and Welfare management and then b) describe the potential benefits and feasibility of an industry-specific HSW management system?**

Equestrian includes work and non-work environments. Some Health, Safety and Welfare regulators or Sports Governance expect the industry to manage and reduce *risk.* I would like to know what we can do to improve HSW management and if the equestrian industry would benefit from a best-practice equestrian industry-specific Health, Safety and Welfare management system?

- What are three key elements to improve HSW for the equestrian industry to maintain best-practice standards?
- What would the industry's ideal equestrian-specific Health, Safety, and Welfare management system include?
- Describe the benefits and feasibility of this for your <specific equestrian sector>?
- What does <specific equestrian sector> need as one-sector in industry, to adopt and implement an equestrian specific Health, Safety and Welfare management system?
- Describe any disadvantages or barriers for <specific equestrian sector>?
- Who are <specific equestrian sector> key influencers who would endorse an equestrian specific Health, Safety and Welfare management system?
- What process does <specific equestrian sector> use to adopt and implement change management?
- Do you have any other comments about Health, Safety and Welfare management in the industry today or looking forward to the future?
